# Effect of Dynamic High-Pressure Microfluidization on the Quality of Not-from-Concentrate Cucumber Juice

**DOI:** 10.3390/foods13132125

**Published:** 2024-07-03

**Authors:** Zhiwei Zhang, Meiyue Zhang, Zhenhong Gao, Yuying Cheng, Xinyi Yang, Shuaixue Mu, Kunsheng Qu

**Affiliations:** 1 School of Biotechnology and Food Science, Tianjin University of Commerce, Tianjin 300134, China; 18698007250@163.com (M.Z.); gzh9290@163.com (Z.G.); 15866561201@163.com (Y.C.); m17856075055@163.com (X.Y.); msx17737430641@163.com (S.M.); qukunsheng@126.com (K.Q.); 2 Tianjin Key Laboratory of Food Biotechnology, School of Biotechnology and Food Science, Tianjin University of Commerce, Tianjin 300134, China

**Keywords:** dynamic high-pressure microfluidization, cucumber juice, not-from-concentrate, volatile flavor substances

## Abstract

The effects of dynamic high-pressure microfluidization (DHPM at 400 MPa) and heat treatment (HT) on the microbial inactivation, quality parameters, and flavor components of not-from-concentrate (NFC) cucumber juice were investigated. Total aerobic bacteria, yeasts and molds were not detected in the 400 MPa-treated cucumber juice. Total phenolic content increased by 16.2% in the 400 MPa-treated cucumber juice compared to the control check (CK). The significant reduction in pulp particle size (volume peak decreasing from 100–1000 μm to 10–100 μm) and viscosity increased the stability of the cucumber juice while decreasing the fluid resistance during processing. HT decreased the ascorbic acid content by 25.9% (*p* < 0.05), while the decrease in ascorbic acid content was not significant after 400 MPa treatment. A total of 59 volatile aroma substances were identified by gas chromatography–ion mobility spectrometry (GC-IMS), and a variety of characteristic aroma substances (i.e., valeraldehyde, (*E*)-2-hexenal, (*E*)-2-nonenal, and (*E*,*Z*)-2,6-nonadienal, among others) were retained after treatment with 400 MPa. In this study, DHPM technology was innovatively applied to cucumber juice processing with the aim of providing a continuous non-thermal processing technology for the industrial production of cucumber juice. Our results provide a theoretical basis for the application of DHPM technology in cucumber juice production.

## 1. Introduction

In recent years, not-from-concentrate (NFC) juice has occupied a considerable share in the consumption of juice in developed countries, and has also become a growing consumption trend in the markets of emerging countries [1]. NFC juice is cleaned and pressed out of fresh fruit juice, and then directly filled after sterilization, which completely retains the original fresh flavor of the fruit. Compared with concentrated juice, NFC juice has higher nutritional value and a more attractive taste, which meets the demand for healthy, natural and safe juice [2,3]. At present, there are NFC apple juice [4], NFC pear juice [5], NFC orange juice [1], etc., on the market.

Cucumber is a globally grown vegetable crop. It is rich in water, contains nutritional functional components, and has a wide range of culinary, therapeutic, and cosmetic applications. Cucumber can be consumed fresh or processed and is popular as a refreshing vegetable [6]. Fresh cucumber juice is favored for its good flavor and low-calorie content. Studies on clear cucumber juice processed by ultrafiltration [7], frozen concentrated cucumber juice [8], and cucumber juice beverages [9] have been published. However, there are few reports on NFC cucumber juice.

Traditional thermal processing methods are gradually being replaced by some emerging technologies because they destroy the heat-sensitive components of fruit juices, leading to a decrease in their quality. Dynamic high-pressure microfluidization (DHPM) is a new continuous non-thermal processing technology applicable to fluids [10]. Under high pressure, fluids are forced to pass through narrow gaps, which generates high shear stress, high turbulence, and cavity effects [11]. This results in the efflux of small molecules from the particles and an increase in nutrients. DHPM has the advantages of low processing temperatures, short time, and continuous operation [12]. DHPM is mainly concerned with altering the viscosity characteristics of the fluid to undergo a physical change and has been effective for the inactivation of microorganisms, the modulation of enzymatic activities, and the improvement of the functional properties of food ingredients [13].

Currently, DHPM has been applied in a variety of food matrices. DHPM retained most of the initial sensory and nutritional qualities of fresh juice and produced high-quality wine [14]. The application of DHPM in carrot juice can improve the carotenoid content and stability, thus providing consumers with desirable high-quality carrot juice [15]. DHPM can reduce the particle size of yam juice, and it has a good effect on killing the harmful microorganisms in yam juice [16]. However, studies on the effect of DHPM treatment on the quality of NFC cucumber juice have not yet been conducted.

In this study, we investigated the effects of DHPM and heat treatment on the microbial inactivation, quality parameters, and characteristic flavor components of NFC cucumber juice and determined their optimal processing techniques. Our aim was to apply DHPM, a non-thermal continuous processing technology, to the cucumber juice production industry, so as to solve the problem of the quality deterioration of processed cucumber juice. This study provides technical guidelines for the commercial application of DHPM technology in NFC cucumber juice processing.

## 2. Materials and Methods

### 2.1. Materials

Fresh ripe cucumbers were purchased from a local farmer market (Tianjin, China). Plate count and rose bengal agar (Solarbio Technology Co., Ltd., Beijing, China). Methanol, gallic acid, Folin–Ciocalteu reagents and L-ascorbic acid (McLean Biochemical Technology Co., Ltd., Shanghai, China).

### 2.2. Preparation of Samples

**Figure 1:**
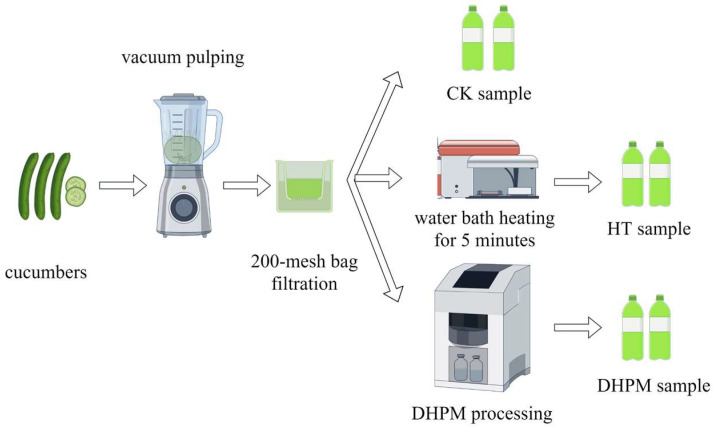
The processing flowchart of cucumber juice.

The processing flowchart of cucumber juice was illustrated in Figure 1.

#### 2.2.1. Preparation of Cucumber Juice

As shown in Figure 1, 3 kg of cucumbers of uniform size and color and without visual defects or mechanical damage were selected, rinsed, and cut into 2–3 cm pieces. They were placed into a vacuum pulping machine (HR3752/00, Philips, Amsterdam, The Netherlands) for processing. The entire slurry was filtered through a 200-mesh filter bag and a portion of the collected cucumber juice was used as the control check (CK). Another part of the slurry was heated for 5 min in a boiling water bath, filtered, and labeled as heat-treated juice (HT). The remaining juice was subjected to DHPM [17]. The above procedure was repeated three times and the treated cucumber juice was stored at 4 °C for no more than 3 days until analysis.

#### 2.2.2. DHPM

The remaining slurry from 2.2.1 was divided equally into three samples and subjected to DHPM at 200, 300, and 400 MPa, respectively. The flow rate of the DHPM machine (ZH600-THW18, Wenzhou Binyi Technology Machinery, Wenzhou, China) was 18 L/h, and the processing temperature was 25 °C. Processed samples (500 mL) were rapidly cooled in an ice water bath and stored at 4 °C until analyzed, not exceeding 3 days of storage [16].

### 2.3. Microbial Analysis

The total aerobic bacteria in cucumber juice were determined by plate count with reference to the National Standard of the People’s Republic of China, GB 4789.2-2022 [18]. All prepared media, reagents and utensils were sterilized in an autoclave sterilizer (SX-500, TOMY, Timex Yitoa Laboratory Equipment, Shanghai, China). The cucumber juice samples were serially diluted with 0.85% sterile NaCl, and 1.0 mL of each dilution was applied to 15 mL agar plates, and then inverted and placed in an incubator at 36 ± 1 °C for 48 h. Yeast and molds were determined using rose bengal agar, with reference to the National Standard of the People’s Republic of China, GB 4789.15-2016 [19], and the samples were coated with the plates. After the samples were coated, the plates were set positively and incubated in an incubator at 28 ± 1 °C for up to 5 days. The number of microbial colonies was converted to log10 value. All samples were measured three times.

### 2.4. Determination of Titratable Acidity, pH, and Total Soluble Solids

Titratable acidity (TA) and pH were measured using an automatic titrator (ET18, Mettler Toledo potentiometric titrator, Zurich, Switzerland) at 20 °C. TA was determined by diluting 5 mL of sample with 0.1 mol/L NaOH and 50 mL of distilled water to the end point (pH 8.2 ± 0.1, phenolphthalein indicator changed to light pink), and the results were expressed as citric acid (%). Total soluble solids (TSS) content in °Brix was measured using an Abbe refractometer (WYA-2S, Shanghai Jingke Industrial Co., Ltd., Shanghai, China) at 20 °C [20]. The above indexes were repeated three times for each sample.

### 2.5. Color

The color of cucumber juice was measured using a portable colorimeter (Chroma Meter CR400, Konica Minolta, Tokyo, Japan) against a white background using the Commission International de I’Eclairage CIE color system. The color parameters L*, a*, and b* were recorded and repeated three times for each sample. The total color difference ∆*E* was calculated according to Equation (1) [21].

(1) $$△E=\sqrt{{\left(L^{*}-L_{0}^{*}\right)}^{2}+{\left(a^{*}-a_{0}^{*}\right)}^{2}+{\left(b^{*}-b_{0}^{*}\right)}^{2}}$$

where ∆*E* is the total color difference, L*, a*, and b* are the luminance, red-greenness and yellow-blueness of the sample to be tested, and $L_{0}^{*}$, $a_{0}^{*}$, and $b_{0}^{*}$ are the luminance, red-greenness and yellow-blueness of the CK, respectively.

### 2.6. Chlorophyll Content

The chlorophyll content was determined by Zhao et al. [22]’s method, with appropriate modifications. A total of 2 mL of the sample was taken in a 50 mL centrifuge tube, 10 mL of extraction solution (acetone–anhydrous ethanol=1:1) was added, and the sample was shaken with a vortex mixer for 1 h. After that, the sample was allowed to stand at room temperature protected from light for 5 h. The sample was filtered through a 0.45 μm organic membrane and analyzed by a UV–visible spectrophotometer (HITACHI U-5100, Tianjin, China) at 645 nm and 663 at room temperature. The determination was repeated three times for each sample. The chlorophyll content was determined using the following equation:

(2) $$\mathrm{Chla}=12.71\times A_{663}-2.59\times A_{645}$$

(3) $$\mathrm{Chlb}=22.88\times A_{645}-4.67\times A_{663}$$

where Chla is the content of chlorophyll a, Chlb is the content of chlorophyll b, and A_663_ and A_645_ are the absorbance at 663 and 645 nm, respectively.

### 2.7. Ascorbic Acid

The 2,6-dichloroindophenol method was used to determine the ascorbic acid content of cucumber juice with reference to theNational Standard of the People’s Republic of China, GB 5009.86-2016 [23]. Accurately weigh 20 g of cucumber juice in a beaker, transfer it to a 100 mL volumetric flask with 2% metaphosphoric acid solution, fix, shake well, and filter it for measurement. The filtrate was titrated with 2,6-dichloroindophenol solution until the solution was pink in color for 15 s without fading, and a blank titration was carried out at the same time, which was repeated three times for each sample. The final results were all expressed as mg/100 mL cucumber juice.

### 2.8. Determination of Total Phenolic

Total phenolic content was determined by the Folin–Ciocalteu method [24]. Take 30 g of cucumber juice and add 30 mL of 80% methanol solution, perform ultrasonic extraction at room temperature for 15 min, centrifugation at 12,000 r for 15 min, then take the supernatant and dilute it 10 times, and that is the crude extract of the polyphenols. The Folin–Ciocalteu reagent was mixed with distilled water 1:9, 0.4 mL of polyphenol crude extract was mixed with 2 mL of diluted Folin–Ciocalteu reagent, and 1.8 mL of 7.5% Na_2_CO_3_ solution was added; the reaction was carried out in the dark at room temperature for 1 h, and the absorbance value at 765 nm was determined by spectrophotometer. Gallic acid was used as the standard to make the standard curve, and the total phenolic content was expressed as gallic acid equivalent mg GAE/100 g of cucumber juice. Each sample was repeated three times.

### 2.9. Stability Indicators

#### 2.9.1. Turbidity

Cucumber juice (10 mL) was centrifuged at 4000 rpm for 10 min, and the absorbance of the supernatant (2 mL) at 660 nm was measured using a spectrophotometer [3], using distilled water as the blank. Repeat the measurement 3 times for each sample.

#### 2.9.2. Particle Size

A Masterizer 3000 laser particle size analyzer (Malvern Instruments, Malvern, UK) was used to determine the particle size distribution (PSD) of cucumber juice samples. After sufficient washing, the samples were dispersed drop by drop in a stirring beaker containing 400 mL of distilled water until the shade reached 5% [25]. Volume–mean particle size (D [4,3]) and area–mean particle size (D [3,2]) were calculated using the software (Microtrac-Bluewave, version 21 CFR Part 11) that came with the instrument. The determination was repeated three times for each sample.

#### 2.9.3. Rheological Properties

Viscosity measurements were performed using a rotational rheometer (MCR301, Anton Paar, Austrian) with a flat plate rotor (49.983 mm in diameter), with a measurement gap of 1 mm and a water circulation system to control the assay temperature at 25 ± 1 °C. Briefly, the cucumber juice sample (2.5 mL) was placed between the fixture and flat plate rotor, and the shear rate was increased from 0.1 s^−1^ to 100 s^−1^ in a steady-state mode [26]. The determination was repeated three times for each sample.

### 2.10. GC-IMS Analysis

Cucumber juice odor was analyzed using gas-phase ion mobility spectrometry (FlavourSpec, G.A.S, Dortmund, Germany), [27,28]. The chromatographic column type was MXT-5 (15 m × 0.53 mm ID × 1 μm). Cucumber juice (2 mL) was placed in a 20-mL headspace vial and incubated at 50 °C for 20 min, with an injection volume of 500 μL at 85 °C. The total analysis time was 30 min. The carrier gas was high-purity N_2_ (purity ≥99.999%) in a non-separated mode, with a flow rate of 2 mL/min for 2 min, increasing to 10 mL/min for 8 min and 75 mL/min for 20 min. The analytes were separated by elution at 50 °C and ionized in an ion mobility spectrometry (IMS) ionization chamber, containing a tritium ionization source in the positive ionization mode at 6.5 KeV. The drift gas was N_2_, with a flow rate of 150 mL/min, and the temperature of the drift tube was 45 °C.

The retention index (RI) of the volatile compounds in the cucumber juice samples was calculated using the ortho-ketone C4–C9 [29,30]. Volatile organic compounds were characterized by comparing the RI and ion drift time with the standards in the gas chromatography GC-IMS NIST library. GC–IMS data were analyzed using the Gallery Plot and Reporter plug-ins provided by VOCal, and principal component analysis (PCA) was performed for different treatments.

### 2.11. Statistical Analysis

All results are the averages of 3 separate batches of cucumber juice, and each sample was analyzed in triplicate. The results were expressed as mean value ± standard deviation (SD) [15]. All experiments were replicated three times, and data were analyzed using the SPSS version 25 software. One-way analysis of variance (ANOVA) was used to determine significance at 95% confidence level. Origin PRO 2021 and GraphPad Prism 9.5 were used for plotting.

## 3. Results and Discussion

### 3.1. Microbiological Analysis

The effect of different treatments on the inactivation of microorganisms in NFC cucumber juice is listed in Table 1. Total aerobic bacteria, yeast and molds were not detected after both heat treatment and 400 MPa DHPM treatment. As the homogenization pressure increased, the number of total aerobic bacteria decreased, and the number of yeast and molds had dropped below the detection limit at 200 MPa.

HT, as the most commonly used sterilization method in juice processing, is undoubtedly effective. DHPM, as a new non-thermal sterilization technology, produces various mechanical effects during its action, such as strong shear, high-frequency vibration, high-speed impact, and transient pressure drop and cavitation, which lead to high fragmentation of particles and cell membranes [11]. Under the above mechanical forces, the integrity of the microbial cell wall in cucumber juice was destroyed, and with the increase in pressure, the cell structure was torn more and finally completely destroyed, leading to the complete inactivation of the microorganisms [31,32].

DHPM significantly killed the microorganisms in the yam juice and the number of microorganisms decreased with increasing pressure [16]. Liu et al. [33] showed that there were fewer and fewer microorganisms in pear juice as the temperature increased, while the higher the homogenizing pressure at a certain temperature, the better the microbial extinguishing effect. This is consistent with the effect of the two processing methods on microbial inactivation in this study. In this study, microbial counts could be reduced below the detection limit under HT and 400 MPa conditions, and therefore, cucumber juice samples treated with both were used for subsequent testing.

### 3.2. TA, pH, and TSS

**Figure 2:**
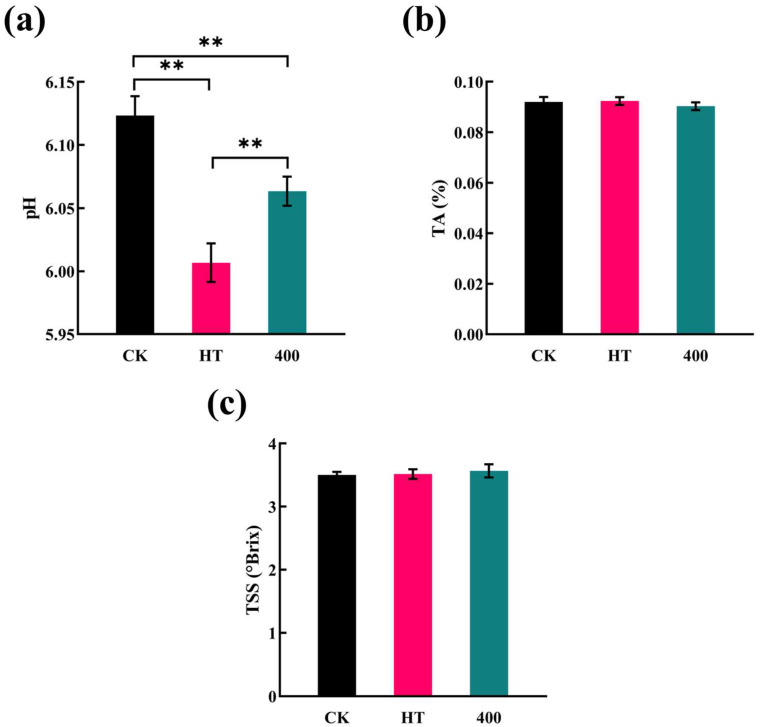
Effect of HT and DHPM treatment at 400 MPa on the pH, TA, and TSS of NFC cucumber juice. (**a**) pH, (**b**) TA, and (**c**) TSS. CK, no treatment; HT, heat treatment; 400, 400 MPa DHPM-treated sample. * *p* < 0.05, statistical differences; ** *p* < 0.01, significant statistical differences.

As shown in Figure 2a, the initial pH of cucumber juice was approximately 6.13, which was significantly decreased (*p* < 0.05) after HT and 400 MPa treatments. It can be seen that pH decreased less by 400 MPa treatment than HT treatment. 

The decrease in pH after 400 MPa DHPM treatment was mainly due to the destructive effect of the mechanical action produced by this technique on the cucumber pulp, which allowed some of the acid to flow out [34]. Similar results were reported in pear juice [33], kiwi juice [35], and peach juice [36]. A decrease in the pH of cucumber juice after HT treatment may be due to a change in the buffer salts in the system, resulting in a change in pH; the exact reason for this has not been clearly reported in any study [37], and further research is needed.

As shown in Figure 2b,c, the TA and TSS values of cucumber juice treated with HT and 400 MPa DHPM fluctuated compared to CK; however, these changes were not significant (*p* > 0.05). Similarly, in pomegranate juice, TA and TSS were not affected by pressure and high-temperature pasteurization [38]. Wellala et al. [39] reported that DHPM treatment had no significant effect on the TSS and TA contents of blended juice. The minimal changes in TSS and TA observed in this study highlight the non-destructive nature of DHPM on the covalent bonds within the cucumber juice matrix [40].

### 3.3. Color Analysis and Chlorophyll a b

Color is an important indicator of the quality of NFC cucumber juice, and processing technology can affect the color change of the juice. The color change parameters of cucumber juice samples after HT and DHPM treatments are presented in Table 2. The L* (brightness) of CK and HT treatments were found to be 27.73 and 24.39, respectively. The L* was observed to be 25.57 after 400 MPa DHPM treatment. There was a decrease in the L* after both HT and DHPM treatments. Similarly, a* (red-green) and b* (yellow-blue) were also affected by both treatments, showing a decrease in green and an increase in yellow, and these changes were more pronounced in the HT treatment. ∆E for the HT and 400 MPa treatments were 3.90 and 2.44, respectively, which were both greater than 2, indicating that these treatments produced color changes that were visible to the naked eye but were superior to the HT treatment for DHPM.

The increase in pressure during DHPM treatment inevitably leads to an increase in temperature, which leads to non-enzymatic browning [41,42,43], implying that even a brief exposure to high temperatures can trigger non-enzymatic browning [44], which can lead to color changes in cucumber juice. Several studies have been conducted to show that DHPM treatment affects the color of juice. Karacam et al. [45] showed that by increasing the amount of homogenization and pressure, brightness L* decreased but b* and ∆*E* increased. All the L* values of mango juice decreased after high pressure homogenization, while a* increased with increasing pressure, temperature and number of homogenizations [46]. DHPM treatment was associated with a decrease in a* in turbid apple juice, which was less reddish than the initial product, while 300 MPa treatment was associated with an increase in b* [47].

**Figure 3:**
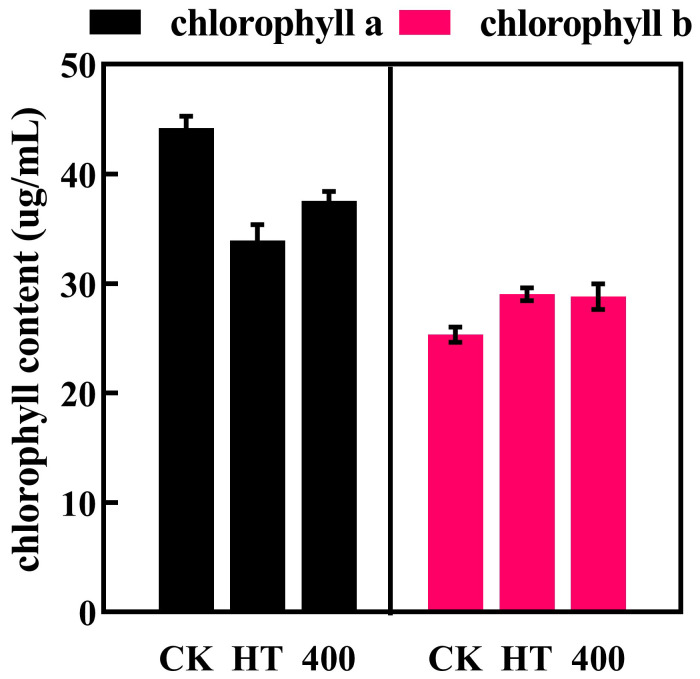
Effect of HT and DHPM treatment on chlorophyll a and b content of NFC cucumber juice.

Chlorophyll is the main pigment responsible for the green color of cucumber juice. The contents of chlorophyll a and chlorophyll b in cucumber juice are shown in Figure 3. Compared with CK, HT and DHPM treatment decreased the chlorophyll a content, with HT resulting in a larger decrease of 23.2% (from 44.22 to 33.94 ug/mL), which was consistent with the decrease in the a* value of the color index. The change in chlorophyll b content was opposite to that in chlorophyll a, i.e., significantly increased by 14.6% after HT (from 25.34 to 29.04 ug/mL), and its content was increased in cucumber juice treated with DHPM treatment at 400 MPa (from 25.34 to 28.82 ug/mL).

An increase in temperature due to elevated pressure is unavoidable during DHPM treatment [43]. It is well known that chlorophyll is an extremely unstable pigment that is highly susceptible to destruction at higher temperatures, which is mainly due to the significant increase in the activity of chlorophyll-degrading enzymes (including chlorophyll-degrading enzymes and chlorophyll-degrading peroxidases) after heat stress treatments, which accelerated the degradation of chlorophyll [48]. In addition, Wang et al. [49] found that the stability of spinach chlorophyll was affected by pH, and an elevated pH would improve the stability of chlorophyll to a certain extent. This is consistent with our findings that pH tends to decrease after HT and DHPM treatments, thus reducing chlorophyll stability to some extent.

### 3.4. Ascorbic Acid and Total Phenols

Ascorbic acid is highly sensitive to oxidative and thermal degradation during fruit and vegetable juice processing, and is often used as a marker of product quality deterioration. Polyphenols are important bioactive components in cucumber juice, and their content is used to measure product quality [50].

**Figure 4:**
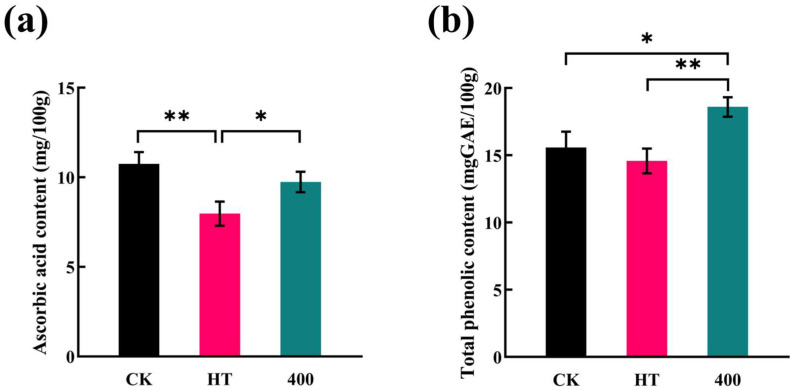
Effect of HT and DHPM treatment on the contents of ascorbic acid and total phenols in NFC cucumber juice: (**a**) ascorbic acid content and (**b**) total phenolic content. * *p* < 0.05, statistical differences; ** *p* < 0.01, significant statistical differences.

As shown in Figure 4a, the ascorbic acid content of CK samples was 10.75 mg/100 g. This is similar to that reported by Shan et al. [51]. HT significantly reduced the ascorbic acid content by 25.9%, whereas DHPM did not result in a significant reduction. Figure 4b represents the variation in total phenolic content in cucumber juice. The total phenolic content of 400 MPa treatment was 18.6 mg GAE/100 g, which increased by 16.2% compared to that of CK (15.58 mg GAE/100 g). HT reduced the total phenolic content; however, the change was not significant. 

DHPM treatment increased the phenolic content, which was attributed to the high mechanical forces that directly induced the hydrolysis and depolymerization of the complexes to form pre-existing phenolic compounds, while at the same time, the process disrupted the cell wall and increased the solubility of the polyphenols, leading to an increase in the polyphenol content [31,35]. The total phenolic content of composite pear juice increased significantly with increasing pressure after DHPM treatment [33]. DHPM treatment increased the total phenolic content of blackcurrant juice by 5% [44]. Polyphenols are important constituents of fruits and vegetables, and elevated total phenolic content has a positive effect on the color and taste of fruit and vegetable juices. However, regarding ascorbic acid, its stability is poorer than that of other polyphenolic compounds [52]. It is more sensitive to temperature and more easily oxidized. During homogenization, the pressure and temperature increase; despite the rapid passage of the cucumber juice through the homogenization chamber, instantaneous pyrolysis occurs. This process is also accompanied by the entry of oxygen, leading to the oxidation of ascorbic acid. DHPM reduced the ascorbic acid content of the kiwifruit juice, and the content decreased with increasing homogenization pressure [43]. The decrease in ascorbic acid content in blackcurrant juice was more pronounced at an inlet temperature of 20 °C and a pressure of 220 MPa [44].

### 3.5. Turbidity, PSD, and Rheological Properties

**Figure 5:**
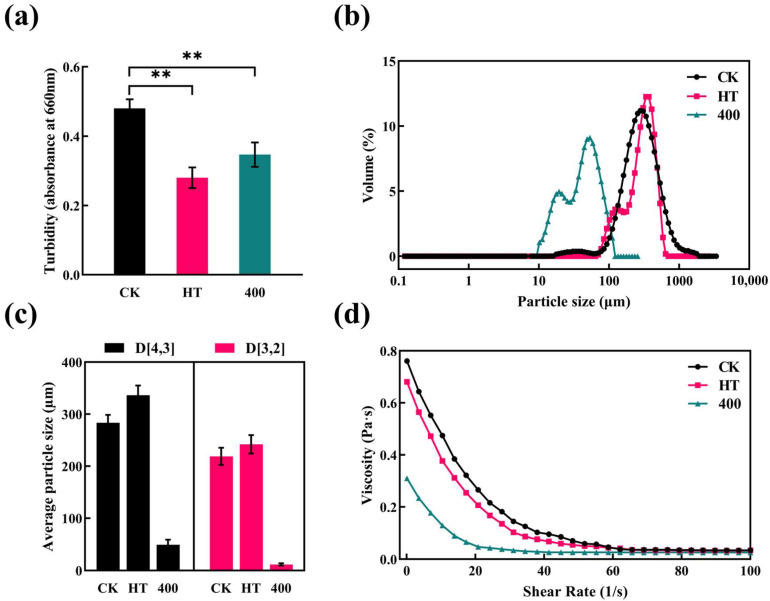
Effect of HT and DHPM treatment on turbidity, particle size, and rheological properties of NFC cucumber juice: (**a**) turbidity, (**b**) particle size, (**c**) average particle size, and (**d**) viscosity. * *p* < 0.05, statistical differences; ** *p* < 0.01, significant statistical differences.

The turbid state of fruit and vegetable juices is the result of the dispersion of insoluble particles, such as pectin, protein, lipids, and cellulose. Turbidity is an indicator of the stability of NFC fruit and vegetable juices [15]. The effect of HT and DHPM on the turbidity of the cucumber juice is shown in Figure 5a. The highest turbidity was observed for CK, while the turbidity of cucumber juice samples treated with both heat treatment and DHPM decreased to varying degrees. The main reason for the decrease in turbidity of cucumber juice after 400 MPa DHPM treatment is that mechanical forces, such as shear and cavitation, disrupt the shape and structure of the cucumber juice, resulting in smaller particle diameters [53]. Smaller suspended particles allow more light to pass through, resulting in lower absorbance, which is directly related to the turbidity of the sample [17]. After HT treatment, turbidity was significantly reduced. Pectin, fibers, proteins, polysaccharides, and other macromolecular substances in the cucumber juice thermally coagulate and flocculate due to heat [44]; the weight of the flocculated material increases, and settling occurs owing to gravity, which results in a decrease in turbidity. Similarly, Wang et al. [54] reported significantly lowering the turbidity of peach juice treated by DHPM, with it gradually decreasing with increases in homogenization pressure and number of passes. 

PSD characterizes the degree of pulp destruction during fruit and vegetable juice processing and is closely related to the stability and rheological properties of fruit and vegetable juices. As expected, DHPM treatment reduced the particle size of cucumber juice pulp. In Figure 5b, the particle size of the untreated sample was concentrated in the 100–1000 μm range with a single-peak distribution; whereas, after 400 MPa treatment, the particle size was significantly reduced and was mainly concentrated in the 10–100 μm range with a double-peak distribution. During DHPM, the excessive pressure resulted in the full rupture of pulp particles into debris and the formation of more fine particles [53]. Liu et al. [33] and Leite et al. [55] also showed that the particle size of both DHPM-treated composite pear juice and frozen orange juice concentrate decreased significantly, and the PSD changed from a single peak to a double peak. Compared with that of CK, the PSD of HT cucumber juice shifted to the right, indicating a larger particle size, which could be attributed to an increased temperature that promotes protein denaturation and aggregation, thus increasing the particle size [54].

From Figure 5c, both the D [4,3] and D [3,2] of the cucumber juice decreased after 400 MPa treatment. Compared with CK, D [4,3] decreased by 82.3%, and D [3,2] decreased by 94.5%, with the reduction in D [3,2] being significantly greater than that in D [4,3]. D [4,3] is mainly affected by large particles, while D [3,2] is more sensitive to small particles [56], and the value of D [3,2] is larger due to the fact that more small particles are produced after DHPM treatment. Yu et al. [57] also reported a significant decrease in D [4,3] and D [3,2] after DHPM treatment in taro pulp. After HT, the increases of D [4,3] and D [3,2] were 18.7% and 10.5%, respectively.

Figure 5d shows that with increasing shear rate, the viscosity gradually decreased, and shear thinning occurred. Thus, the cucumber juice behaved as a typical non-Newtonian fluid, showing pseudoplastic behavior. This result is consistent with those observed in DHPM-treated NFC orange juice [56] and composite pear juice [33]. The cucumber juice with reduced viscosity was more fluid and closer to clear juice in terms of taste.

At low shear rates ranging from 0.1–40 s^−1^, the viscosity of cucumber juice decreased at a faster rate, whereas at the range of 40–100 s^−1^, the viscosity change was not obvious and showed a Newtonian fluid state. At 0.1 s^−1^, the viscosity of 400 MPa-treated cucumber juice decreased by 59.2% compared with that of the CK sample. When the homogenizing pressure was increased, pectin, which plays a decisive role in the viscosity of the cucumber juice, was broken down, and the intermolecular forces between pectin molecules were weakened [58]. In addition, the suspended particles of pectin in cucumber juice cause charge neutralization, leading to colloidal aggregation, and all of these changes lead to a decrease in the viscosity of cucumber juice. In this study, it can be concluded that DHPM can reduce the viscosity of NFC cucumber juice by decreasing the particle size and changing the size of pectin molecules [56], thus reducing the fluid resistance and energy consumption in the processing industry.

### 3.6. GC-IMS Analysis

#### 3.6.1. Volatile Components

**Figure 6:**
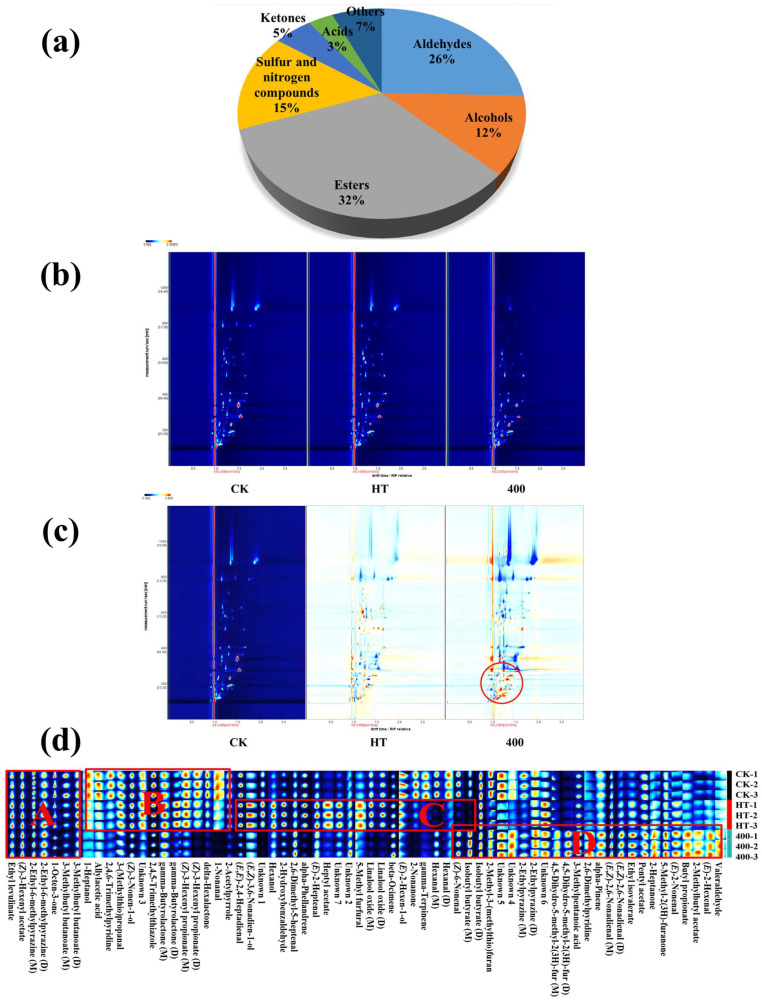
GC-IMS analysis of volatile components in NFC cucumber juice. (**a**) Proportion of different categories of volatile compounds. (**b**) Top view of 3D topography. (**c**) Comparison of the differences between the two treatments. (**d**) Fingerprints of volatile components of cucumber juice.

Fifty-nine volatiles were identified by GC-IMS, including 15 aldehydes, 7 alcohols, 19 esters, 9 sulfur–nitrogen compounds, 3 ketones, 2 acids, and 4 other compounds, and the percentages of each category are shown in Figure 6a. Table 3 shows all the volatile substances, including (*E*,*Z*)-2,6-nonadienal, (*E*,*Z*)-3,6-nonadien-1-ol, (*E*)-2-nonenal, (*Z*)-6-nonenal, 1-nonanal, and hexanal, and many other substances with “cucumber, grassy, refreshing, and fruity” flavors. Based on previous studies [9,51,59], the above substances were identified as the characteristic aroma substances of cucumber juice.

#### 3.6.2. Two-Dimensional Mapping

The top view of the 3D topography is shown in Figure 6b. The red vertical line at the horizontal coordinate 1.0 represents the reactive ion peak (RIP, normalized); the vertical coordinate represents the retention time (s) of the GC; and the horizontal coordinate represents the ion migration time. Each point on the right side of the RIP represents a volatile organic substance, and the color represents the concentration of the substance, with white indicating a lower concentration and red indicating a higher concentration. The drift time of volatile substances in cucumber juice ranged from 1–1.75, and the retention time ranged from 100–1000 s.

To compare the differences more intuitively, the spectrum of CK was selected as a reference, and the spectrum of the other samples was deducted from the reference. If the two volatile organic compounds were the same, the deducted background would be white; otherwise, red indicates that the content of the substance was higher than the reference, while blue indicates that the content of the substance was lower than the reference [60]. As shown in Figure 6c, there was a significant difference in the volatile matter of cucumber juice between the two treatments. Compared with those in CK and HT, the volatile substances in cucumber juice subjected to 400 MPa DHPM had a significant increasing trend at the red circle, which corresponds to the substances within region D in Figure 6d.

#### 3.6.3. Analysis of GC-IMS Fingerprints of NFC Cucumber Juice

To clarify the changes in the volatile components in cucumber juice after HT and DHPM, fingerprint profiles were compared. A fingerprint profile line was generated for each peak in the GC-IMS two-dimensional spectra, to identify the respective characteristic peak regions of the different samples. Each horizontal row represents all the selected signal peaks in the cucumber juice of one treatment, and each column represents the signal peaks of the same volatile flavor substance [61].

From Figure 6d, the fingerprints can be divided into four regions. Region A is the common peak region of the volatile compounds in the cucumber juice of the three treatments, with no difference in content. Ethyl levulinate, (*Z*)-3-hexenyl acetate, 2-ethyl-6-methylpyrazine, 1-octen-3-one, and 3-methylbutyl butanoate are found in Region A, exhibiting fruity, nutty, and baking aromas. Region B shows a significant difference between DHPM, CK, and HT, indicating that the content of volatiles in this region was reduced after DHPM treatment, including substances such as 2,4,6-trimethylpyridine, gamma-butyrolactone, and (*Z*)-3-hexenyl propionate. In this region, 2,4,5-trimethylthiazole (earthy flavor) and 3-(methylthio)propanal appeared after HT treatment as volatile sulfides (steaming flavor, broth flavor), which are the source of undesirable flavors in juices [62], and were significantly reduced by DHPM treatment. Region C indicates that HT treatment results in the presence of linalool oxide (burnt, earthy flavor) and 5-methyl furfural (roasted, almond flavor, a product of the meladic reaction), while the content of the characteristic odorants of cucumber, (*Z*)-6-nonenal (fresh, cucumber flavor) and hexanal (grassy flavor) were significantly reduced. It has been shown that melon juice produces a strong ripening off-flavor during thermal processing, leading to a severe reduction in aroma quality [63]. However, the contents of (*E*)-2-heptenal and (*E*,*Z*)-3,6-nonadien-1-ol increased significantly, resulting in a stronger and fresher cucumber flavor. Finally, in region D, the DHPM treatment increased the content of 25 volatiles, including (*E*)-2-nonenal, (*E*,*Z*)-2,6-nonadienal, (*E*)-2-hexenal, and valeraldehyde, which gave the cucumber juice a fresh odor. At the same time, from 3-methylpentanoic acid (sour, herbal flavor) and 2-methyl-3-(methylthio)furan (stinky odor), unpleasant odors are produced. DHPM has been shown to reduce some of the volatile aromas in sake, but treatment with HT is even less effective [37].

Compared with HT, DHPM contributed more to the flavor substances of cucumber juice. Under high mechanical shear stress, the internal odor molecules were released or decomposed, and the overall performance showed that the release was greater than the degradation, suggesting that DHPM can be applied in the production of NFC cucumber juice instead of HT treatment.

#### 3.6.4. Principal Component Analysis

**Figure 7:**
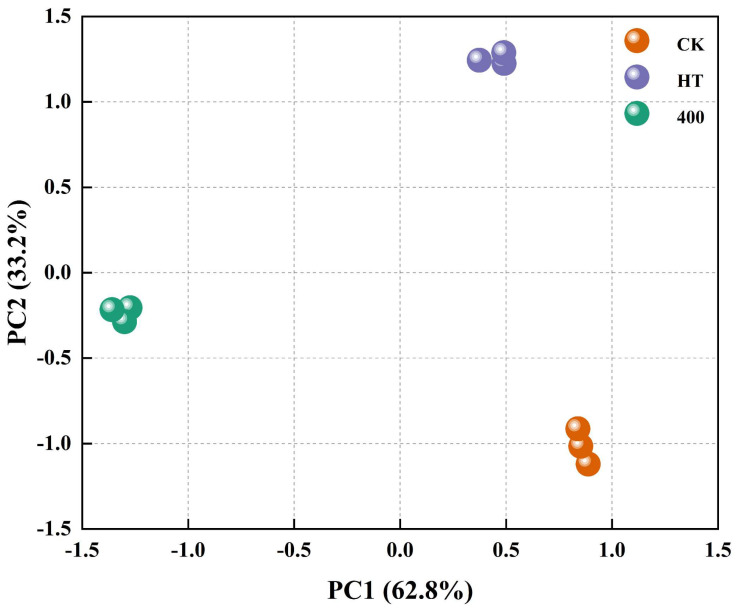
PCA of volatile formation in NFC cucumber juice subjected to HT and DHPM.

Figure 7 shows the PCA plot of NFC cucumber juice samples subjected to no treatment, HT, and DHPM. A close distance between the samples indicates similar aroma characteristics and vice versa [64]. Generally, PCA is the preferred separation model when the cumulative contribution of PC1 and PC2 reaches 60% [65]. As shown in Figure 7, the contribution rate of PC1 was 62.8%, while that of PC2 was 33.2%. The cumulative variance contribution rate was 96%, confirming the reliability of the PCA results. The PCA results showed that both HT and DHPM could alter the aroma composition of NFC cucumber juice.

## 4. Conclusions

In this study, DHPM technology was applied to the processing of NFC cucumber juice, which is a new combination. DHPM, as a new non-thermal processing technology, has the advantage of applying dynamic pressure to cucumber juice compared with high-pressure processing, which can realize the continuous production of cucumber juice in the industry. Compared with previous studies, this study used a higher homogenization pressure to inactivate all the microorganisms in one treatment at 400 MPa, avoiding the degradation of cucumber juice quality caused by multiple treatments at low pressure. Compared with CK, there was no significant change in TA and TSS after 400 MPa DHPM treatment, reflecting the non-destructive nature of this technology on the covalent bonds in the cucumber juice matrix; the total phenol content was increased by 16.2%, which improved the nutritive value; the particle size of the pulp was significantly reduced, which improved the suspension stability of the cucumber juice; the viscosity was greatly reduced after the treatment, which in turn reduced the fluid resistance in the industrial processing; and a total of 59 volatile substances were identified using GC-IMS, among which a variety of cucumber characteristic aroma components were still retained. Compared with HT, cucumber juice treated at 400 MPa showed less loss of ascorbic acid and less color browning. The loss of heat-sensitive components caused by the processing technology of juice production is inevitable, and more in-depth research on this issue is needed in the future to promote the further development of the juice industry.

## Figures and Tables

**Table 1:** Effect of different treatments on microorganisms of NFC cucumber juice.

Microbial Species	CK Sample	HT Sample	200 MPa DHPM-Treated Sample	300 MPa DHPM-Treated Sample	400 MPa DHPM-Treated Sample
Total aerobic bacteria	4.56 ± 0.13 ^a^	ND	2.49 ± 0.12 ^b^	1.66 ± 0.08 ^c^	ND
Yeast and molds	3.37 ± 0.08 ^a^	ND	ND	ND	ND

Unit, log CFU/mL. Values represent the mean ± standard error (*n* = 3). Columns with different superscript letters are significantly different (*p* < 0.05). ND, not detected (detection limit < 1 CFU/mL); CK, no treatment; HT, heat treatment; DHPM, dynamic high-pressure microfluidization.

**Table 2:** Effect of HT and DHPM treatment on the color of NFC cucumber juice.

Treatment Condition	L*	a*	b*	∆*E*
CK	27.73 ± 0.22 ^a^	−2.66 ± 0.12 ^a^	3.54 ± 0.08 ^c^	Control
HT	24.39 ± 0.28 ^d^	−2.17 ± 0.18 ^c^	5.49 ± 0.19 ^a^	3.90 ± 0.47 ^a^
400-MPa DHPM	25.57 ± 0.32 ^c^	−2.20 ± 0.2 ^c^	4.57 ± 0.11 ^b^	2.44 ± 0.14 ^b^

Values represent the mean ± standard error (*n* = 3). Columns with different superscript letters are significantly different (*p* < 0.05).

**Table 3:** Identification of volatile substances in NFC cucumber juice using GC-IMS.

No.	Compound	CAS#	Formula	MW	RI	Rt [s]	Dt [a.u.]	Peak Intensity		
								CK	HT	400
1	(*E*)-2-Nonenal	C18829566	C_9_H_16_O	114.2	863.9	118.9	1.12	2906.36 ± 33.57 ^c^	4270.49 ± 9.61 ^b^	4948.25 ± 233.29 ^a^
2	(*E*)-2-Hexen-1-ol	C928950	C_6_H_12_O	100.2	866.1	120.83	1.16	1537.16 ± 50.02 ^a^	1150.46 ± 57.8 ^b^	787.65 ± 58.49 ^c^
3	5-Methyl-2(3H)-furanone	C591128	C_5_H_6_O_2_	98.1	878.1	131.74	1.12	1849.13 ± 28.89 ^b^	1881.91 ± 56.72 ^b^	2478.6 ± 132.42 ^a^
4	(*E*)-2-Hexenal	C6728263	C_6_H_10_O	98.1	881.1	134.66	1.2	126.52 ± 5.84 ^c^	217.85 ± 8.16 ^b^	244.43 ± 2.78 ^a^
5	2-Methylbutyl acetate	C624419	C_7_H_14_O_2_	130.2	881.6	135.17	1.29	39.6 ± 4.81 ^c^	50.49 ± 1.26 ^b^	96.44 ± 10.67 ^a^
6	2-Heptanone	C110430	C_7_H_14_O	114.2	888.4	141.96	1.25	540.44 ± 9.62 ^c^	742.99 ± 24.23 ^b^	957.91 ± 9.95 ^a^
7	Allylacetic acid	C591800	C_5_H_8_O_2_	100.1	890.3	143.89	1.14	704.46 ± 26.45 ^a^	548.87 ± 12.77 ^b^	483.8 ± 17.64 ^c^
8	Butyl propionate	C590012	C_7_H_14_O_2_	130.2	891.9	145.63	1.29	92.97 ± 9.1 ^b^	90.09 ± 5.41 ^b^	161.55 ± 1.15 ^a^
9	2,6-Dimethylpyridine	C108485	C_7_H_9_N	107.2	895.6	149.95	1.1	286.16 ± 10.88 ^b^	249.31 ± 42.89 ^b^	514.65 ± 13.87 ^a^
10	Pentyl acetate	C628637	C_7_H_14_O_2_	130.2	911.4	170.63	1.31	708.04 ± 16.12 ^b^	636.12 ± 3.6 ^c^	944.58 ± 37.47 ^a^
11	3-(Methylthio)propanal	C3268493	C_4_H_8_OS	104.2	912.5	172.16	1.4	753.05 ± 26.95 ^b^	806.07 ± 34.32 ^a^	578.76 ± 22.06 ^c^
12	Valeraldehyde	C110623	C_5_H_10_O	122.2	919.5	182.19	1.26	137.59 ± 5.36 ^a^	119.37 ± 7.6 ^b^	146.99 ± 5.78 ^a^
13	gamma-Butyrolactone (M)	C96480	C_4_H_6_O_2_	86.1	919.7	182.5	1.08	555.05 ± 9.68 ^b^	646.08 ± 10.05 ^a^	530.52 ± 14.66 ^c^
14	gamma-Butyrolactone (D)	C96480	C_4_H_6_O_2_	86.1	921.5	185.18	1.31	1781.82 ± 17.37 ^b^	3325.87 ± 43.26 ^a^	1251.36 ± 38.44 ^c^
15	2-Ethylpyrazine (D)	C13925003	C_6_H_8_N_2_	108.1	923.5	188.25	1.2	452.83 ± 2.39 ^b^	379.99 ± 8.91 ^c^	549.71 ± 26.53 ^a^
16	(*E*,*Z*)-3,6-Nonadien-1-ol	C56805233	C_9_H_16_O	96.1	926	192.08	1.39	1913.27 ± 51.67 ^b^	2595.77 ± 54.64 ^a^	1974.83 ± 37.44 ^b^
17	alpha-Pinene	C80568	C_10_H_16_	136.2	928.4	195.86	1.2	351.74 ± 3.53 ^b^	349.81 ± 10.54 ^b^	415.6 ± 9.38 ^a^
18	2-Ethylpyrazine (M)	C13925003	C_6_H_8_N_2_	108.1	929.6	197.79	1.11	408.22 ± 29.24 ^a^	216.29 ± 8.12 ^c^	320.28 ± 2.04 ^b^
19	Ethyl isovalerate	C108645	C_7_H_14_O_2_	130.2	940.6	216.37	1.25	220.72 ± 4.64 ^c^	346.16 ± 15.85 ^b^	445.73 ± 8.65 ^a^
20	Isobutyl butyrate (M)	C539902	C_8_H_16_O_2_	144.2	944.1	222.7	1.34	2994.96 ± 127.9 ^b^	676.63 ± 15.92 ^c^	6409.55 ± 255.32 ^a^
21	2-Methyl-3-(methylthio)furan	C63012975	C_6_H_8_OS	128.2	945.1	224.53	1.09	2130.94 ± 34.55 ^a^	1362.25 ± 32.6 ^c^	1970.48 ± 45.89 ^b^
22	Isobutyl butyrate (D)	C539902	C_8_H_16_O_2_	144.2	950.3	234.14	1.36	4492.39 ± 55.91 ^a^	4012.62 ± 21.27 ^c^	4295.78 ± 95.29 ^b^
23	(*E*)-2-Heptenal	C18829555	C_7_H_12_O	112.2	955.9	245.18	1.26	700.37 ± 13.48 ^c^	1198.95 ± 2.67 ^a^	1118.65 ± 24.51 ^b^
24	4,5-Dihydro-5-methyl-2(3H)-fur (D)	C108292	C_5_H_8_O_2_	100.1	956.4	246.11	1.41	367.28 ± 15.71 ^c^	560.91 ± 16.68 ^b^	1624.85 ± 73.36 ^a^
25	4,5-Dihydro-5-methyl-2(3H)-fur (M)	C108292	C_5_H_8_O_2_	100.1	956.5	246.28	1.13	311.4 ± 17.14 ^b^	286.42 ± 5.25 ^c^	880.27 ± 19.52 ^a^
26	5-Methyl furfural	C620020	C_6_H_6_O_2_	110.1	957.2	247.74	1.48	134.44 ± 4.67 ^b^	251.77 ± 2.88 ^a^	120.24 ± 9.85 ^b^
27	Hexanal (D)	C66251	C_6_H_12_O	106.1	961.6	256.66	1.16	263.53 ± 18.16 ^a^	127.15 ± 8.73 ^c^	152.61 ± 3.65 ^b^
28	3-Methylpentanoic acid	C105431	C_6_H_12_O_2_	116.2	967.2	268.76	1.27	679.57 ± 1.51 ^b^	718.23 ± 12.11 ^b^	997.25 ± 49.08 ^a^
29	1-Heptanol	C111706	C_7_H_16_O	116.2	969.4	273.46	1.41	232.33 ± 15.25 ^a^	182.83 ± 12.6 ^b^	255.01 ± 30.35 ^a^
30	1-Octen-3-one	C4312996	C_8_H_14_O	126.2	974	284.01	1.27	4131.04 ± 24.61 ^a^	3498.12 ± 68.05 ^b^	2462.42 ± 45.47 ^c^
31	Hexanal (M)	C66251	C_6_H_12_O	106.1	984.8	309.98	1.5	536.49 ± 19.08 ^a^	102.68 ± 7.51 ^d^	354.53 ± 25.87 ^c^
32	(*Z*)-6-Nonenal	C2277192	C_9_H_16_O	128.2	984.9	310.27	1.16	1070.97 ± 11.52 ^a^	327.59 ± 5.56 ^c^	990.24 ± 44 ^b^
33	2-Ethyl-6-methylpyrazine (M)	C13925036	C_7_H_10_N_2_	122.2	996.5	341.19	1.18	6215.5 ± 52.26 ^a^	6112.79 ± 18.09 ^a^	5493.32 ± 56.56 ^b^
34	2,4,5-Trimethylthiazole	C13623115	C_6_H_9_NS	127.2	996.8	342.04	1.52	20,699.43 ± 255.71 ^a^	15,497.9 ± 236.43 ^b^	9065.59 ± 226.34 ^c^
35	(*E*,*Z*)-2,6-Nonadienal (D)	C557482	C_9_H_14_O	120.2	998.4	346.86	1.24	287.87 ± 6.45 ^c^	459.01 ± 14.39 ^b^	751.34 ± 6.74 ^a^
36	2,4,6-Trimethylpyridine	C108758	C_8_H_11_N	121.2	1003.2	362.23	1.58	768.36 ± 43.26 ^a^	468.52 ± 28.72 ^b^	325.72 ± 21.12 ^c^
37	2-Ethyl-6-methylpyrazine (D)	C13925036	C_7_H_10_N_2_	122.2	1003.3	362.68	1.64	1160.68 ± 76.02 ^a^	1231.34 ± 24.95 ^a^	1215.92 ± 26.31 ^a^
38	(*Z*)-3-Hexenyl acetate	C3681718	C_8_H_14_O_2_	142.2	1004	364.97	1.33	3285.69 ± 81.98 ^b^	3500.57 ± 33.28 ^a^	3602.44 ± 50.5 ^a^
39	(*E*,*E*)-2,4-Heptadienal	C4313035	C_7_H_10_O	110.2	1014.3	400.34	1.15	1902.11 ± 26.94 ^a^	1783.25 ± 27.64 ^b^	960.3 ± 30.2 ^c^
40	alpha-Phellandrene	C99832	C_10_H_16_	136.2	1015.3	403.86	1.69	1890.17 ± 84.69 ^b^	2275.26 ± 246.12 ^a^	526.29 ± 26.74 ^c^
41	(*Z*)-3-Nonen-1-ol	C10340235	C_9_H_18_O	144.2	1015.6	405.17	1.34	2539.04 ± 45.5 ^a^	2595.74 ± 87.74 ^a^	1679.86 ± 46.29 ^b^
42	Hexanol	C111273	C_6_H_14_O	122.2	1019.8	420.63	1.12	334.69 ± 17.84 ^c^	593.83 ± 4.48 ^a^	492.45 ± 22.03 ^b^
43	beta-Ocimene	C13877913	C_10_H_16_	136.2	1040.2	505.3	1.67	1748.84 ± 139.53 ^c^	2426.53 ± 95.07 ^b^	3162.82 ± 462.14 ^a^
44	(*E*,*Z*)-2,6-Nonadienal (M)	C557482	C_9_H_14_O	120.2	1041	508.84	1.25	4023.9 ± 158.91 ^b^	4471.85 ± 81.32 ^b^	5143.62 ± 336.08 ^a^
45	Heptyl acetate	C112061	C_9_H_18_O_2_	158.2	1041.6	511.95	1.46	45.64 ± 3.57 ^c^	177.78 ± 3.25 ^a^	57.62 ± 6.87 ^b^
46	2-Hydroxybenzaldehyde	C90028	C_7_H_6_O_2_	122.1	1041.8	512.63	1.15	586.28 ± 9.54 ^b^	1121.83 ± 9.1 ^a^	574.95 ± 14.45 ^b^
47	2,6-Dimethyl-5-heptenal	C106729	C_9_H_16_O	140.2	1051.6	559.89	1.16	857.25 ± 32.4 ^c^	1136.07 ± 9.92 ^b^	1357.04 ± 56.73 ^a^
48	3-Methylbutyl butanoate (D)	C106274	C_9_H_18_O_2_	158.2	1052.8	566.31	1.42	1032.34 ± 82.74 ^b^	1240.77 ± 27.47 ^a^	872.28 ± 15.93 ^c^
49	gamma-Terpinene	C99854	C_10_H_16_	136.2	1053.6	570.25	1.71	249.48 ± 14.52 ^a^	90.45 ± 15.08 ^b^	71.59 ± 4.97 ^b^
50	3-Methylbutyl butanoate (M)	C106274	C_9_H_18_O_2_	158.2	1061	609.24	1.41	1162.15 ± 79.23 ^a^	781.36 ± 37.9 ^b^	588.25 ± 27.25 ^c^
51	Ethyl levulinate	C539888	C_7_H_12_O_3_	144.2	1064.4	628.55	1.2	3356.38 ± 99.27 ^b^	3845.04 ± 85.59 ^a^	3120.62 ± 56.96 ^c^
52	Linalool oxide (M)	C60047178	C_10_H_18_O_2_	170.3	1070	660.85	1.27	858.81 ± 18.33 ^b^	967.21 ± 45.01 ^a^	269.25 ± 6.48 ^c^
53	Linalool oxide (D)	C60047178	C_10_H_18_O_2_	170.3	1077.9	709.35	1.82	378.45 ± 19.03 ^b^	455.12 ± 16.11 ^a^	163.47 ± 8.73 ^c^
54	delta-Hexalactone	C823223	C_6_H_10_O_2_	114.1	1089.2	785.37	1.17	4067.24 ± 123.03 ^a^	2546.55 ± 192.38 ^b^	807.94 ± 25.67 ^c^
55	2-Nonanone	C821556	C_9_H_18_O	142.2	1090	791.25	1.86	899.15 ± 34.19 ^a^	338.31 ± 40.32 ^b^	123.37 ± 12.47 ^c^
56	2-Acetylpyrrole	C1072839	C_6_H_7_NO	109.1	1090.7	796.16	1.48	2335.48 ± 116.51 ^a^	1504.98 ± 121.37 ^b^	713.05 ± 15.95 ^c^
57	(Z)-3-Hexenyl propionate (M)	C33467742	C_9_H_16_O_2_	156.2	1102.2	882.58	1.37	5901.68 ± 32.4 ^a^	5888.42 ± 54.88 ^a^	2329.99 ± 16.85 ^b^
58	(*Z*)-3-Hexenyl propionate (D)	C33467742	C_9_H_16_O_2_	156.2	1102.3	883.47	1.9	5384.49 ± 81.49 ^a^	4386.57 ± 294.21 ^b^	499.12 ± 2.71 ^c^
59	1-Nonanal	C124196	C_9_H_18_O	142.2	1103.8	895.33	1.93	2881.48 ± 66.19 ^a^	1917.83 ± 197.68 ^b^	165.17 ± 9.81 ^c^

CK, no treatment; HT, heat treatment; 400, 400 MPa DHPM-treated sample. MW, molecular mass; RI, the retention index (experimental value); Rt, retention time in s; Dt, drift time in a.u. The M and D inside parentheses represent the monomer and dimer forms, respectively. Columns with different superscript letters differ significantly (*p* < 0.05) in peak intensity.

## Data Availability

The original contributions presented in the study are included in the article, further inquiries can be directed to the corresponding author.

## References

[1] Zhang J., Liu H., Sun R., Zhao Y., Xing R., Yu N., Deng T., Ni X., Chen Y. (2022). Volatolomics approach for authentication of not-from-concentrate (NFC) orange juice based on characteristic volatile markers using headspace solid phase microextraction (HS-SPME) combined with GC-MS. Food Control.

[2] Teixeira J.C., Ribeiro C., Simoes R., Alegria M.J., Mateus N., de Freitas V., Perez-Gregorio R., Soares S. (2023). Characterization of the Effect of a Novel Production Technique for ’Not from Concentrate’ Pear and Apple Juices on the Composition of Phenolic Compounds. Plants.

[3] Wang H., Yuan J., Chen L., Ban Z., Zheng Y., Jiang Y., Jiang Y., Li X. (2022). Effects of Fruit Storage Temperature and Time on Cloud Stability of Not from Concentrated Apple Juice. Foods.

[4] Kosiński J., Cywińska-Antonik M., Szczepańska-Stolarczyk J., Jasińska U.T., Woźniak Ł., Kaniewska B., Marszałek K. (2024). Application of an Electromagnetic Field for Extending the Shelf-Life of Not from Concentrate (NFC) Apple Juice. Appl. Sci..

[5] Gan X., Ma Q., Wang L., Liu W., Chen Z., Wang W., Wang J., Mu J. (2023). Physicochemical, sensory characterisation and volatile components of 16 NFC pear juice. J. Food Meas. Charact..

[6] Saad A.M., Mohamed A.S., El-Saadony M.T., Sitohy M.Z. (2021). Palatable functional cucumber juices supplemented with polyphenols-rich herbal extracts. LWT.

[7] Liu F., Zhang X., Zhao L., Wang Y., Liao X. (2016). Potential of high-pressure processing and high-temperature/short-time thermal processing on microbial, physicochemical and sensory assurance of clear cucumber juice. Innov. Food Sci. Emerg. Technol..

[8] Mohd Rosli N.N.H., Harun N.H., Abdul Rahman R., Ngadi N., Samsuri S., Amran N.A., Safiei N.Z., Ab Hamid F.H., Zakaria Z.Y., Jusoh M. (2022). Preservation of total phenolic content (TPC) in cucumber juice concentrate using non-thermal Progressive Freeze Concentration: Quantitative design characteristics and process optimization. J. Clean. Prod..

[9] Zhao L., Wang S., Liu F., Dong P., Huang W., Xiong L., Liao X. (2013). Comparing the effects of high hydrostatic pressure and thermal pasteurization combined with nisin on the quality of cucumber juice drinks. Innov. Food Sci. Emerg. Technol..

[10] Zheng J., Wang N., Huang S., Kan J., Zhang F. (2021). In vitro digestion and structural properties of rice starch modified by high methoxyl pectin and dynamic high-pressure microfluidization. Carbohydr. Polym..

[11] Chen Y., Sun Y., Meng Y., Liu S., Ding Y., Zhou X., Ding Y. (2023). Synergistic effect of microfluidization and transglutaminase cross-linking on the structural and oil-water interface functional properties of whey protein concentrate for improving the thermal stability of nanoemulsions. Food Chem..

[12] Wang N., Wang R., Xing K., Huang Z., Elfalleh W., Zhang H., Yu D. (2024). Microfluidization of soybean protein isolate-tannic acid complex stabilized emulsions: Characterization of emulsion properties, stability and in vitro digestion properties. Food Chem..

[13] Levy R., Okun Z., Shpigelman A. (2020). High-Pressure Homogenization: Principles and Applications Beyond Microbial Inactivation. Food Eng. Rev..

[14] Morata A., Guamis B. (2020). Use of UHPH to Obtain Juices With Better Nutritional Quality and Healthier Wines With Low Levels of SO_2_. Front. Nutr..

[15] Jin X., Huang L., Wang H. (2024). Effects of dynamic high pressure microfluidization on the physical and chemical properties of carrot (*Daucus carota* L.). Bangladesh J. Bot..

[16] Liu M., Wang R., Li J., Zhang L., Zhang J., Zong W., Mo W. (2021). Dynamic high pressure microfluidization (DHPM): Physicochemical properties, nutritional constituents and microorganisms of yam juice. Czech J. Food Sci..

[17] Abliz A., Liu J., Mao L., Yuan F., Gao Y. (2021). Effect of dynamic high pressure microfluidization treatment on physical stability, microstructure and carotenoids release of sea buckthorn juice. LWT.

[18] (2022). Food Microbiological Examination: Determination of Aerobic Plate Count.

[19] (2016). Food Microbiological Examination: Enumeration of Moulds and Yeasts.

[20] Mandha J., Shumoy H., Matemu A.O., Raes K. (2023). Characterization of fruit juices and effect of pasteurization and storage conditions on their microbial, physicochemical, and nutritional quality. Food Biosci..

[21] Tighiceanu C., Bulai E.R., Iatcu O.C., Dulucheanu C., Nemtoi A. (2023). Effect of Vegetable Juices on Properties of Two Resin Composites Used for Dental Caries Management. Medicina.

[22] Zhao L., Qin X., Han W., Wu X., Wang Y., Hu X., Ling J., Liao X. (2018). Novel application of CO_2_-assisted high pressure processing in cucumber juice and apple juice. LWT.

[23] (2016). Determination of Ascorbic Acid in Foods.

[24] Rizvi N.B., Fatima A., Busquets R., Khan M.R., Ashraf S., Khan M.S., Oz F. (2023). Effect of the Media in the Folin-Ciocalteu Assay for the Analysis of the Total Phenolic Content of Olive Products. Food Anal. Methods.

[25] Zhang W., Li Y., Jiang Y., Hu X., Yi J. (2023). A Novel Strategy to Improve Cloud Stability of Orange-Based Juice: Combination of Natural Pectin Methylesterase Inhibitor and High-Pressure Processing. Foods.

[26] Han S.H., Zhu J.K., Shao L., Yue C.H., Li P.Y., Bai Z.Y., Luo D.L. (2024). Effects of Ultrasonic Treatment on Physical Stability of Lily Juice: Rheological Behavior, Particle Size, and Microstructure. Foods.

[27] Zhou Y., Wang D., Duan H., Zhou S., Guo J., Yan W. (2023). Detection and analysis of volatile flavor compounds in different varieties and origins of goji berries using HS-GC-IMS. LWT.

[28] Xi B.-N., Zhang J.-J., Xu X., Li C., Shu Y., Zhang Y., Shi X., Shen Y. (2024). Characterization and metabolism pathway of volatile compounds in walnut oil obtained from various ripening stages via HS-GC-IMS and HS-SPME-GC–MS. Food Chem..

[29] Liu H., Yu Y., Zou B., Yu Y., Yang J., Xu Y., Chen X., Yang F. (2023). Evaluation of Dynamic Changes and Regularity of Volatile Flavor Compounds for Different Green Plum (Prunus mume Sieb. et Zucc) Varieties during the Ripening Process by HS-GC–IMS with PLS-DA. Foods.

[30] Rong Y., Xie J., Yuan H., Wang L., Liu F., Deng Y., Jiang Y., Yang Y. (2023). Characterization of volatile metabolites in Pu-erh teas with different storage years by combining GC-E-Nose, GC–MS, and GC-IMS. Food Chem. X.

[31] Huang X., Li C., Xi J. (2023). Dynamic high pressure microfluidization-assisted extraction of plant active ingredients: A novel approach. Crit. Rev. Food Sci. Nutr..

[32] Reverter-Carrión L., Sauceda-Gálvez J.N., Codina-Torrella I., Hernández-Herrero M.M., Gervilla R., Roig-Sagués A.X. (2018). Inactivation study of Bacillus subtilis, Geobacillus stearothermophilus, Alicyclobacillus acidoterrestris and Aspergillus niger spores under Ultra-High Pressure Homogenization, UV-C light and their combination. Innov. Food Sci. Emerg. Technol..

[33] Liu Y., Liao M., Rao L., Zhao L., Wang Y., Liao X. (2022). Effect of ultra-high pressure homogenization on microorganism and quality of composite pear juice. Food Sci. Nutr..

[34] Guo X., Chen M., Li Y., Dai T., Shuai X., Chen J., Liu C. (2020). Modification of food macromolecules using dynamic high pressure microfluidization: A review. Trends Food Sci. Technol..

[35] Patrignani F., Mannozzi C., Tappi S., Tylewicz U., Pasini F., Castellone V., Riciputi Y., Rocculi P., Romani S., Caboni M.F. (2019). (Ultra) High Pressure Homogenization Potential on the Shelf-Life and Functionality of Kiwifruit Juice. Front. Microbiol..

[36] Yildiz G. (2019). Application of ultrasound and high-pressure homogenization against high temperature-short time in peach juice. J. Food Process Eng..

[37] Arakawa G.Y., Yokoi K.J. (2023). Application of multiple ultra-high-pressure homogenization to the pasteurization process of Japanese rice wine, sake. J. Biosci. Bioeng..

[38] Benjamin O., Gamrasni D. (2020). Microbial, nutritional, and organoleptic quality of pomegranate juice following high-pressure homogenization and low-temperature pasteurization. J. Food Sci..

[39] Wellala C.K.D., Bi J., Liu X., Liu J., Lyu J., Zhou M., Marszałek K., Trych U. (2020). Effect of high pressure homogenization combined with juice ratio on water-soluble pectin characteristics, functional properties and bioactive compounds in mixed juices. Innov. Food Sci. Emerg. Technol..

[40] Ravichandran C., Jayachandran L.E., Kothakota A., Pandiselvam R., Balasubramaniam V.M. (2023). Influence of high pressure pasteurization on nutritional, functional and rheological characteristics of fruit and vegetable juices and purees-an updated review. Food Control.

[41] Rolandelli G., Favre L.C., Mshicileli N., Vhangani L.N., Farroni A.E., van Wyk J., Buera M.d.P. (2021). The complex dependence of non-enzymatic browning development on processing conditions in maize snacks. LWT.

[42] Sajib M., Undeland I. (2020). Towards valorization of herring filleting by-products to silage 2.0: Effect of temperature and time on lipid oxidation and non-enzymatic browning reactions. LWT.

[43] Wang X.Y., Wu S.H., Zong W. (2018). Comparison of the Influence of Dynamic High-Pressure Microfluidization and Conventional Homogenization on the Quality of Kiwi Fruit Juice. Appl. Eng. Agric..

[44] Kruszewski B., Zawada K., Karpiński P. (2021). Impact of High-Pressure Homogenization Parameters on Physicochemical Characteristics, Bioactive Compounds Content, and Antioxidant Capacity of Blackcurrant Juice. Molecules.

[45] Karacam C.H., Sahin S., Oztop M.H. (2015). Effect of high pressure homogenization (microfluidization) on the quality of Ottoman Strawberry (F. Ananassa) juice. LWT Food Sci. Technol..

[46] Zhou L., Guan Y., Bi J., Liu X., Yi J., Chen Q., Wu X., Zhou M. (2017). Change of the rheological properties of mango juice by high pressure homogenization. LWT Food Sci. Technol..

[47] Sauceda-Gálvez J.N., Codina-Torrella I., Martinez-Garcia M., Hernández-Herrero M.M., Gervilla R., Roig-Sagués A.X. (2021). Combined effects of ultra-high pressure homogenization and short-wave ultraviolet radiation on the properties of cloudy apple juice. LWT.

[48] Rossi S., Burgess P., Jespersen D., Huang B. (2017). Heat-Induced Leaf Senescence Associated with Chlorophyll Metabolism in Bentgrass Lines Differing in Heat Tolerance. Crop Sci..

[49] Wang R., Wang T., Zheng Q., Hu X., Zhang Y., Liao X. (2012). Effects of high hydrostatic pressure on color of spinach purée and related properties. J. Sci. Food Agric..

[50] Xu X., Pan J., He M., Tian H., Qi X., Xu Q., Chen X. (2019). Transcriptome profiling reveals key genes related to astringency during cucumber fruit development. 3 Biotech.

[51] Shan N., Gan Z., Nie J., Liu H., Wang Z., Sui X. (2020). Comprehensive Characterization of Fruit Volatiles and Nutritional Quality of Three Cucumber (Cucumis sativus L.) Genotypes from Different Geographic Groups after Bagging Treatment. Foods.

[52] Suárez-Jacobo Á., Saldo J., Rüfer C.E., Guamis B., Roig-Sagués A.X., Gervilla R. (2012). Aseptically packaged UHPH-treated apple juice: Safety and quality parameters during storage. J. Food Eng..

[53] He X.-H., Luo S.-J., Chen M.-S., Xia W., Chen J., Liu C.-M. (2020). Effect of industry-scale microfluidization on structural and physicochemical properties of potato starch. Innov. Food Sci. Emerg. Technol..

[54] Wang X., Wang S., Wang W., Ge Z., Zhang L., Li C., Zhang B., Zong W. (2019). Comparison of the effects of dynamic high-pressure microfluidization and conventional homogenization on the quality of peach juice. J. Sci. Food Agric..

[55] Leite T.S., Augusto P.E.D., Cristianini M. (2017). Structural and Rheological Properties of Frozen Concentrated Orange Juice (FCOJ) by Multi-Pass High-Pressure Homogenisation (MP-HPH). Int. J. Food Prop..

[56] Yu W., Cui J., Zhao S., Feng L., Wang Y., Liu J., Zheng J. (2021). Effects of High-Pressure Homogenization on Pectin Structure and Cloud Stability of Not-From-Concentrate Orange Juice. Front. Nutr..

[57] Yu Z.-Y., Jiang S.-W., Cao X.-M., Jiang S.-T., Pan L.-J. (2016). Effect of high pressure homogenization (HPH) on the physical properties of taro ( Colocasia esculenta (L). Schott) pulp. J. Food Eng..

[58] Peng X.-y., Mu T.-h., Zhang M., Sun H.-n., Chen J.-w., Yu M. (2016). Effects of pH and high hydrostatic pressure on the structural and rheological properties of sugar beet pectin. Food Hydrocoll..

[59] Du X., Routray J., Williams C., Weng Y. (2022). Association of Refreshing Perception with Volatile Aroma Compounds, Organic Acids, and Soluble Solids in Freshly Consumed Cucumber Fruit. ACS Food Sci. Technol..

[60] Xuan X., Sun R., Zhang X., Cui Y., Lin X., Sun Y., Deng W., Liao X., Ling J. (2022). Novel application of HS-GC-IMS with PCA for characteristic fingerprints and flavor compound variations in NFC Chinese bayberry (*Myrica rubra*) juice during storage. Lwt.

[61] Telloli C., Tagliavini S., Passarini F., Salvi S., Rizzo A. (2023). ICP-MS triple quadrupole as analytical technique to define trace and ultra-trace fingerprint of extra virgin olive oil. Food Chem..

[62] Cheng Y., Han L., Huang L., Tan X., Wu H., Li G. (2023). Association between flavor composition and sensory profile in thermally processed mandarin juices by multidimensional gas chromatography and multivariate statistical analysis. Food Chem.

[63] Luo D., Pan X., Zhang W., Bi S., Wu J. (2022). Effect of glucose oxidase treatment on the aroma qualities and release of cooked off-odor components from heat-treated Hami melon juice. Food Chem..

[64] Castura J.C., Varela P., Næs T. (2023). Investigating paired comparisons after principal component analysis. Food Qual. Prefer..

[65] Wu Z., Chen L., Wu L., Xue X., Zhao J., Li Y., Ye Z., Lin G. (2015). Classification of Chinese Honeys According to Their Floral Origins Using Elemental and Stable Isotopic Compositions. J. Agric. Food Chem..

